# Solution Conformation of Heparin Tetrasaccharide. DFT Analysis of Structure and Spin–Spin Coupling Constants

**DOI:** 10.3390/molecules23113042

**Published:** 2018-11-21

**Authors:** Miloš Hricovíni, Michal Hricovíni

**Affiliations:** Institute of Chemistry, Slovak Academy of Sciences, 84538 Bratislava, Slovakia; Michal.Hricovini@savba.sk

**Keywords:** heparin tetrasaccharide, solution structure, NMR, DFT, spin-spin coupling constants

## Abstract

Density functional theory (DFT) has provided detailed information on the molecular structure and spin–spin coupling constants of heparin tetrasaccharide (GlcNS,6S-IdoA2S-GlcNS,6S-IdoA2S-OMe) representing the predominant heparin repeating-sequence. The fully optimised molecular structures of two tetrasaccharide conformations (differing from each other in the conformational form of the sulphated iduronic acid residue–one ^1^*C*_4_ and the other ^2^*S*_0_) were obtained using the B3LYP/6-311+G(d,p) level of theory and applying explicit water molecules to simulate the presence of a solvent. The theoretical data provided insight into variations of the bond lengths, bond angles and torsion angles, formations of intra- and intermolecular hydrogen bonds and ionic interactions. Optimised molecular structures indicated the formation of a complex hydrogen bond network, including interresidue and intraresidue bonds. The ionic interactions strongly influence the first hydration shell and, together with hydrogen bonds, play an important role in shaping the 3D tetrasaccharide structure. DFT-derived indirect three–bond proton–proton coupling constants (^3^*J*_H-C-C-H_) showed that the best agreement with experiment was obtained with a weighted average of 67:33 (^1^*C*_4_:^2^*S*_0_) of the IdoA2S forms. Detailed analysis of Fermi-contact contributions to ^3^*J*_H-C-C-H_ showed that important contributions arise from the oxygen lone pairs of neighbouring oxygen atoms. The analysis also showed that the magnitude of diamagnetic spin–orbit contributions are sufficiently large to determine the magnitude of some proton–proton coupling constants. The data highlight the need to use appropriate quantum-chemical calculations for a detailed understanding of the solution properties of heparin oligosaccharides.

## 1. Introduction

The relationship between the molecular structure of carbohydrates and their properties is indispensable for understanding essential processes in glycobiology. Glycosaminoglycans, such as heparin, belong to the widely studied carbohydrate molecules, which play vital roles in blood coagulation, cell differentiation, viral infection or inflammation [1,2,3,4]. The analysis of heparin and heparin oligosaccharide structures, their dynamics and interactions with proteins have, therefore, often been analysed experimentally and theoretically [5,6,7,8,9,10,11]. Heparin, and its oligosaccharides, are structurally rather complex molecules that are composed of repeating disaccharide units comprising a uronate (either β-d-glucuronate or α-l-iduronate) and a hexosamine, 2-amino-2-deoxy α-d-glucose (α-d-glucosamine). The repeating disaccharide can be substituted by *O*- and *N*-sulphate groups biosynthetically, through a series of sulphotransferase enzymes at positions 2-*O*- of the uronate and 6-*O*- (or more rarely, position 3-*O*-) of the glucosamine residue. Furthermore, the glucosamine is predominantly *N*-sulphated, the remainder bearing *N*-acetyl groups and, although none of the substitutions above are made to completion in heparin, predominant residues consisting of GlcNS,6S and IdoA2S do emerge [2,4].

The determination of 3D carbohydrate structures is complicated not only by the presence of furanose/pyranose ring forms, hydroxymethyl group conformation and the formation of hydrogen bonds, but also by the flexibility of the glycosidic linkages [2,9,12,13,14,15,16,17]. However, the presence of sulphate (or acetyl) groups in various positions in heparin-like saccharides brings further complexity and causes considerable structural heterogeneity. In addition, the 2-*O*-sulphated iduronic acid residue (IdoA2S) can adopt various (^1^*C*_4_, ^4^*C*_1_ and ^2^*S*_0_) conformations (unlike most pyranose rings in saccharides), depending on the structure of the neighbouring residues and the type of counterions [18,19]. This leads to additional complications in the analysis of their conformation and dynamics [20,21,22,23]. However, it has become evident that the application of theoretical quantum-chemical methods, such as density functional theory (DFT), together with high-resolution experimental nuclear magnetic resonance (NMR) data, can reveal details of the structure and conformational equilibria in various heparin oligosaccharides [21,24,25,26,27].

**Figure 1:**
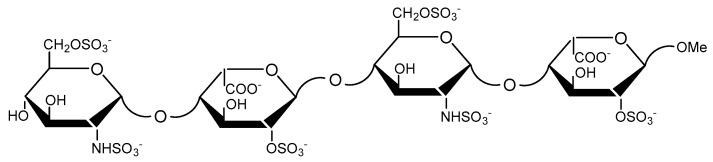
The chemical structure of the heparin tetrasaccharide.

**Figure 2:**
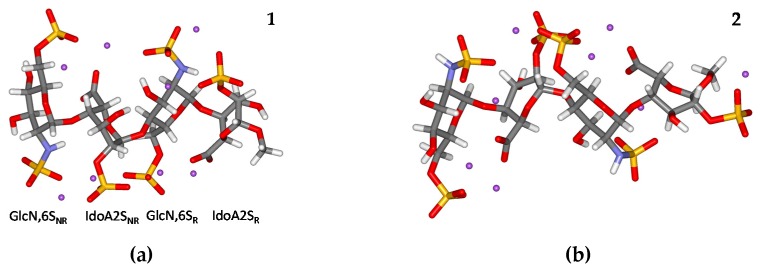
The density functional theory (DFT)–optimised structure of the heparin tetrasaccharide. The two forms, **1** and **2**, have different conformations of the IdoA2S residue. The IdoA2S residues are in the ^1^*C*_4_ conformation in (**a**) and in the ^2^*S*_0_ conformation in (**b**). The GlcNS,6S residues are in the ^4^*C*_1_ conformation. Violet dots represent sodium ions. Solvent (water) molecules are not shown for clarity.

This paper reports results from the detailed analysis of DFT calculations of the heparin tetrasaccharide GlcNS,6S-IdoA2S-GlcNS,6S-IdoA2S-OMe (Figure 1). The molecule was analysed in two forms differing in the conformations (^1^*C*_4_ and ^2^*S*_0_) of the IdoA2S residue: the IdoA2S residues were either in the ^1^*C*_4_ chair form (**1**) in the ^2^*S*_0_ skew form (**2**) (Figure 2). The DFT-derived 3D structures of both **1** and **2** were compared with previous calculations on structurally similar compounds [25,26,27]. In addition, isotropic indirect NMR spin-spin coupling constants were computed, and compared with measured experimental values. Analyses of contributions to spin–spin coupling constants, Fermi–contact contributions and spin-orbit contributions, are also presented.

## 2. Results and Discussion

### 2.1. Geometry

The computed geometry of both heparin tetrasaccharide forms **1** and **2** indicate that bond lengths, bond angles and torsion angles vary with the conformation of the IdoA2S ring (Table 1 and Table 2). Bond lengths and bond angles slightly differ from one another in all residues in **1** and **2** and the variations are comparable to those seen previously [26,27]. For example, the bond lengths at the glycosidic linkages C1-O1 varied up to 0.027 Å; the O1-C4 lengths differed up to 0.035 Å. The values of bond angles did not show any major variations in **1** and **2**; differences up to about 6° were obtained for the C1-O1-C4 bond angle.

The most significant differences between **1** and **2** were seen in torsions angle values. The GlcNS,6S pyranose rings were in the ^4^*C*_1_ chair forms, nevertheless, the ring geometries of **1** and **2** differed from one another to some extent. This is documented by the variations of the heavy atom torsion angles, e.g., O5-C1-C2-C3 (a difference of 16° between **1** and **2** in GlcNS,6S_NR_). Similarly, torsion angles between hydrogens linked to the ring carbons also varied considerably, especially in the GlcNS,6S_NR_ residue (e.g., 26° for H2-C2-C3-H3). The GlcNS,6S_R_ ring geometry was, however, less affected by pseudorotation of the IdoA2S residue. Such changes in the GlcNS,6S residue geometry are comparable with previous DFT calculations [26]. The most significant differences in torsion angle values were computed for the IdoA2S residues due to their different conformations (^4^*C*_1_ vs. ^2^*S*_0_). Apart from the expected differences in the IdoA2S residue, the *φ* and *ψ* torsion angles (*φ* = H1-C1-O1-C4, *ψ* = H4-C4-O1-C1) at the glycosidic linkages also differ somewhat from each other in **1** and **2**. The most significant variations were obtained for *φ* angle (−67° vs. −39°) between the non−reducing end residues (GlcNS,6S_NR_-IdoA2S_NR_) and *ψ* angle (−24° vs. −46°) between the reducing end residues (GlcNS,6S_R_-IdoA2S_R_).

**Figure 3:**
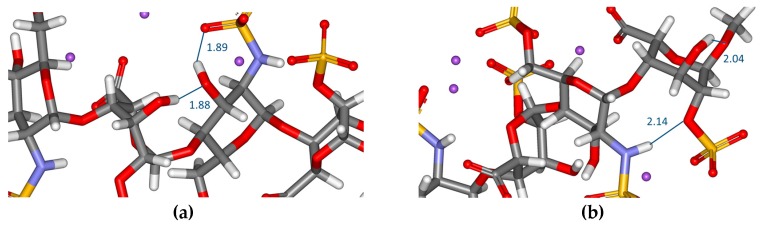
Intramolecular hydrogen bonds in **1**. One interresidue hydrogen bond is between the OH group at C–3 in the IdoA2S ring and the OH group at C–3 and the neighbouring reducing-end GlcNS,6S_R_ residue (computed distance 1.88 Å). (**a**) Second interresidue hydrogen bond (2.14 Å) is between the NH group (GlcNS,6S_R_) and O–2 (2–O–SO_3_^−^ group) in the IdoA2S_R_. (**b**). The other two H-bonds shown in (**a**) and (**b**) are intraresidue.

The DFT calculations have revealed the formation of several intra- and interresidue intramolecular hydrogen bonds (H-bonds). The intraresidue H-bonds were between the OH group at C–3 and the neighbouring NSO_3_^−^ group in GlcNS,6S residues, between OH at C–4 and O–6 in GlcN,6S_NR_ residues or between the OH group at C–3 and O–1 at the reducing end OMe groups in both **1** and **2** in all these cases. The interresidue intramolecular H-bonds (Figure 3) were less frequent; one between the OH group at C–3 in the IdoA2S_NR_ and the OH group at C–3 and the neighbouring GlcNS,6S_R_ residue in **1**. This H-bond was not observed in **2** and instead of the interresidue H-bond, a new intraresidue H-bond was found between the OH group (at C–3) and oxygen in the SO_3_^−^ group linked to C–2. The same types of H-bonds were also observed in the heparin-trisaccharide [26]. In the tetrasaccharide, however, the additional H-bond was observed between the NH group (GlcNS,6S_R_) and O–2 (2–O–SO_3_^−^ group) in the IdoA2S_R_ in **1**. This type of the H-bond was not evident in **2**, but the intraresidue H-bond OH (at C–3)···O (in the SO_3_^−^) (Table 3), similar to that seen in the non-reducing part of the molecule, was observed. These H-bonds are competing with each other and the breakdown of the interresidue H-bonds in **1** and formation of the intraresidue H-bonds in **2** has a strong impact on the conformational equilibrium of the heparin-tetrasaccharide. The data demonstrated that the more stable conformer in the solution (structure **1**, see later discussion) has more interresidue H-bonds than **2** and seems partially stabilised by intramolecular H-bonds.

**Figure 4:**
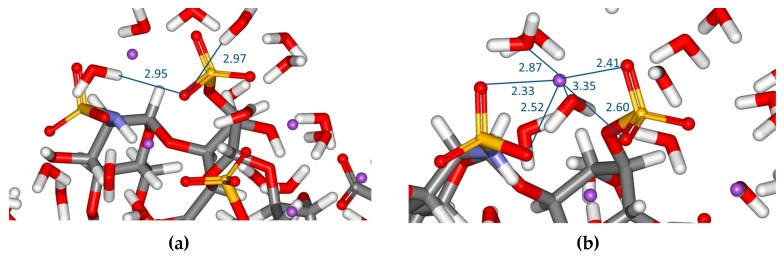
Hydrogen bonds and sodium ion coordination in heparin tetrasaccharide **1**. The computed separations between the pendant groups and water molecules from the first hydration shell X–O···H–O–H are ~2.7–3.1 Å (**a**). Interatomic distances (in Å) refer to oxygen atoms and sodium ions. Oxygen atoms (red) involved in coordination with sodium ion (violet) are displayed as spheres (**b**).

DFT calculations, applying the explicit water model, also allowed the analysis of solute-solvent interactions. Water molecules from the first shell form hydrogen bonds with various saccharide pendant groups. Based on the computed distances between X–O···H–O–H, it is assumed that weak bifurcated hydrogen bonds, both donor and acceptor, are formed in the water solution. (Figure 4a)

Furthermore, it should be noted that heparin and heparin oligosaccharides are strong polyelectrolytes. As seen previously [25], sodium counterions showed a tendency towards 6-fold coordination with oxygen atoms from sulphates, carboxylates and water molecules in both **1** and **2**. The interatomic distances between sulphate or carboxylate oxygen atoms and sodium ions were typically ~2.2 Å and ~2.8 Å, respectively; water oxygen···Na^+^ ion separations were about 2.6–3.6 Å. (Figure 4b) It is known that ion–ion and ion–dipole interactions are stronger than H-bonds and therefore influence the first hydration shell of heparin tetrasaccharide. Thus, apart from intramolecular and intermolecular H-bonds, ionic interactions play an important role in influencing the solution properties of heparin tetrasaccharide.

### 2.2. NMR Spin-Spin Coupling Constants

DFT-computed three-bond proton–proton coupling constants (^3^*J*_H-C-C-H_), computed using the fully optimised geometry of **1** and **2**, are given in Table 4. The magnitudes of coupling constants depended upon torsion angles and varied between 1.19 Hz and 12.32 Hz. The biggest differences between **1** and **2** in the ^3^*J*_H-C-C-H_ magnitudes were in the IdoA2S residues (^1^*C*_4_ and ^2^*S*_0_ forms), e.g., ^3^*J*_H2-C2-C3-H3_ were 2.64 Hz (**1**) and 10.62 Hz (**2**) in the IdoA2S_R_ residue. Noticeable differences were also seen between ^3^*J*_H-C-C-H_ for the same proton pairs in IdoA2S_NR_ and IdoA2S_R_. The computed ^3^*J*_H2-C2-C3-H3_ was 6.17 Hz in the IdoA2S_NR_ unit whereas ^3^*J*_H2-C2-C3-H3_ was 10.62 Hz in the IdoA2S_R_ residue; the differences were also computed for ^3^*J*_H1-C1-C2-H2_ demonstrating ring distortions of the IdoA2S residues. The geometries of these residues differ slightly from one another, although they adopt the ^2^*S*_0_ form in the solution. This indicates that relatively small variations in the ring geometries can result in significantly different ^3^*J*_H-C-C-H_ values and that the ^3^*J*_H-C-C-H_ magnitudes may not be explained reasonably by considering only geometrical factors. As previously mentioned [26], rather complex contributions to ^3^*J*_H-C-C-H_ magnitudes should also be taken into account in heparin tetrasaccharide. Furthermore, ^3^*J*_H-C-C-H_ magnitudes also differed in the GlcNS,6S residues. Thus, the influence of the IdoA residue form upon the GlcNS,6S ring is considerable, as the torsion angle variations were up to 20° (H1-C1-C2-H2 in GlcNS,6S_NR_) and consequently, the ^3^*J*_H-C-C-H_ magnitudes varied: ^3^*J*_H1-C1-C2-H2_ were 4.45 Hz (^1^*C*_4_) and 2.89 Hz (^2^*S*_0_), respectively; even larger differences (9.17 Hz and 11.52 Hz) were obtained for ^3^*J*_H2-C2-C3-H3_.

The weighted average of proton–proton coupling constants <^3^*J*_H-C-C-H_>, using the ratio ^1^*C*_4_:^2^*S*_0_ = 67:33 (**1**:**2**), and the experimental values [28] are listed in the last two columns in Table 4. The data demonstrate that the prevalence (67%) of the chair form in the aqueous solution should be considered to achieve the best fit to the experimental ^3^*J*_H-C-C-H_ values. Most of the weighted average theoretical <^3^*J*_H-C-C-H_> values agreed well with experiment. However, some differences between theory and experiment were observed for the IdoA2S_NR_ residue for <^3^*J*_H2-C2-C3-H3_> and <^3^*J*_H3-C3-C4-H4_>. The geometry of the IdoA2S_NR_ ring, flanked by two GlcNS,6S residues, was more distorted in than the reducing end IdoA2S_R_ residue and led to the pyranose ring flattening and a decrease of the H2-H3 torsion angle (−144.0°) from about 180°. Apart from this geometric distortion, inadequately described delocalisation of the electron density [26] may also be the reason for the differences between the coupling constants. These effects can be examined further by comparing the individual contributions to coupling constants. Fermi contact (FC), spin-dipolar (SD), paramagnetic spin-orbit (PSO) and diamagnetic spin-orbit (DSO) contributions to ^3^*J*_H-C-C-H_ computed at the B3LYP/6-311+(d,p) level are listed in Table 5 (for **1**) and in Table 6 (for **2**). Inspection of the data shows that several coupling constants are different from each other, although they have comparable torsion angles, e.g., ^3^*J*_H3-C3-C4-H4_ (3.27 Hz) in the IdoA2S_NR_ residue in **1** (Table 5, last column) is about 1.8 Hz larger than ^3^*J*_H1-C1-C2-H2_ (1.50 Hz) in the IdoA2S_R_ residue even though the torsion angles are nearly the same (73.3° vs. 72.4°). The corresponding FC terms are 2.71 Hz (IdoA2S_NR_) versus 0.99 Hz (IdoA2S_R_), indicating that the electronic structure must be a strong influence on the magnitude of the FC term. Recent analysis showed [26] that the difference in the FC terms is caused by the presence of oxygen lone pairs in IdoA2S interacting with the electron density of the neighbouring coupled protons. Such interactions can result in the delocalisation of the electron density and, consequently, affect the transmission of the Fermi-contact interaction. Thus, the presence of oxygen lone pairs from the various groups (carboxylate group, the OH groups), located spatially in a different way for diverse coupled proton pairs, results in dissimilar magnitudes of the FC terms.

The weighted average of proton–proton coupling constants <^3^*J*_H-C-C-H_>, using the ratio ^1^*C*_4_:^2^*S*_0_ = 67:33 (**1**:**2**), and the experimental values [28] are listed in the last two columns in Table 4. The data demonstrate that the prevalence (67%) of the chair form in the aqueous solution should be considered to achieve the best fit to the experimental ^3^*J*_H-C-C-H_ values. Most of the weighted average theoretical <^3^*J*_H-C-C-H_> values agreed well with experiment. However, some differences between theory and experiment were observed for the IdoA2S_NR_ residue for <^3^*J*_H2-C2-C3-H3_> and <^3^*J*_H3-C3-C4-H4_>. The geometry of the IdoA2S_NR_ ring, flanked by two GlcNS,6S residues, was more distorted in than the reducing end IdoA2S_R_ residue and led to the pyranose ring flattening and a decrease of the H2-H3 torsion angle (−144.0°) from about 180°. Apart from this geometric distortion, inadequately described delocalisation of the electron density [26] may also be the reason for the differences between the coupling constants. These effects can be examined further by comparing the individual contributions to coupling constants. Fermi contact (FC), spin-dipolar (SD), paramagnetic spin-orbit (PSO) and diamagnetic spin-orbit (DSO) contributions to ^3^*J*_H-C-C-H_ computed at the B3LYP/6-311+(d,p) level are listed in Table 5 (for **1**) and in Table 6 (for **2**). Inspection of the data shows that several coupling constants are different from each other, although they have comparable torsion angles, e.g., ^3^*J*_H3-C3-C4-H4_ (3.27 Hz) in the IdoA2S_NR_ residue in **1** (Table 5, last column) is about 1.8 Hz larger than ^3^*J*_H1-C1-C2-H2_ (1.50 Hz) in the IdoA2S_R_ residue even though the torsion angles are nearly the same (73.3° vs. 72.4°). The corresponding FC terms are 2.71 Hz (IdoA2S_NR_) versus 0.99 Hz (IdoA2S_R_), indicating that the electronic structure must be a strong influence on the magnitude of the FC term. Recent analysis showed [26] that the difference in the FC terms is caused by the presence of oxygen lone pairs in IdoA2S interacting with the electron density of the neighbouring coupled protons. Such interactions can result in the delocalisation of the electron density and, consequently, affect the transmission of the Fermi-contact interaction. Thus, the presence of oxygen lone pairs from the various groups (carboxylate group, the OH groups), located spatially in a different way for diverse coupled proton pairs, results in dissimilar magnitudes of the FC terms.

It should also be noted that the magnitudes of paramagnetic and diamagnetic spin-orbit (DSO) contributions are larger than the Fermi-contact contribution for some ^3^*J*_H-C-C-H_ values in the IdoA2S residues. This is especially noticeable for ^3^*J*_H1-C1-C2-H2_ in the IdoA2S_NR_ residue in **1** where the DSO is 1.76 Hz and PSO is −0.93 Hz (FC = 0.32 Hz) (Table 5, columns 6 and 7). Though the spin-orbit contributions partially cancel each other, the DSO term is so large that it determines the ^3^*J*_H1-C1-C2-H2_ magnitude. Comparable evidence was obtained for the ^3^*J*_H4-C4-C5-H5_ in the IdoA2S_NR_ and, in part also for the same coupling constants in the reducing end iduronate. To illustrate this trend, the individual contributions to H1–H2 coupling constants in monosaccharide IdoA2SOMe and heparin-like oligosaccharides, are listed in Table 7. The DSO terms are dominant and confirm the previous analysis that these terms depend upon geometrical factors due to the contributions of localised molecular orbitals of the adjacent residues. On the other hand, the DSO term is smaller than the FC term due to the absence of contributions of the neighbouring residues in monosaccharide IdoA2SOMe. As mentioned, nearly comparable FC and the DSO terms in the IdoA2S_R_ residue in heparin disaccharide well agrees with the smaller contributions of the single neighbouring unit (GlcNS,6S_NR_). The data thus highlight the difficulties of interpreting correctly spin-spin coupling constants in charged sulphated molecules. This evidence also emphasises that detailed understanding of the solution properties of heparin oligosaccharides can only be achieved by applying rigorous physicochemical approaches, such as DFT calculations combined with high-resolution NMR spectroscopy.

## 3. Materials and Methods

**Geometry optimization** The molecular structure of the heparin tetrasaccharide (GlcNS,6S-IdoA2S-GlcNS,6S-IdoA2S-OMe) has been fully optimised without any constraints applying the ONIOM approach. The B3LYP [29] functional and the 6-311+G(d,p) [30] basis set were applied for the solute and the universal force field (UFF) [31] for the solvent using GAUSSIAN 09 (Wallingford, CT, USA) [32]. The initial positions of the 64 water molecules were based on coordinates of oxygen atoms in water molecules in the published crystal data of sulphated monosaccharides [33,34] and the optimised positions of water molecules in heparin oligosaccharides [26,27]. The geometry optimisation was performed for two conformations of the *L*-IdoA2S residue (^1^*C*_4_, **1**, and ^2^*S*_0_, **2**, Figure 2), whereas the two *D*-GlcNS,6S residues were in the ^4^*C*_1_ conformations. Conformation at the glycosidic linkages *φ* (H1-C1-O1-C4) and *ψ* (H1-C1-O1-C4) for the GlcNS,6S–IdoA2S linkage and positions of counterions were based on the previously published X-ray data for structurally similar compounds [26,27,35]. Hydration of the tetrasaccharide molecules was then performed by the inclusion of explicit water molecules. The isotropic indirect NMR proton–proton spin–spin coupling constant, as well as the individual contributions to coupling constants (i.e., Fermi–contact term, FC; spin–dipolar, SD; paramagnetic spin–orbit, PSO; diamagnetic spin–orbit, DSO contributions), were DFT–computed (B3LYP functional) using the DGDZVP [36] basis set. The theoretical data were then compared with the published experimental data [28].

## 4. Conclusions

In summary, DFT calculations provided a detailed analysis of the molecular structure of the heparin tetrasaccharide in the aqueous solution and enabled interpretation of experimental isotropic indirect NMR spin–spin coupling constants. DFT–computed geometries indicated several differences in bond lengths between intra-ring atoms or at the glycosidic linkages for all residues in **1** and **2**. Differences between **1** and **2** were also found in bond and torsion angle values. Interestingly, the torsion angles at the glycosidic linkages remained, in most cases, comparable regardless of the form of the IdoA2S residue.

The DFT data has provided insight into the formation of intra- and intermolecular H-bonds and ionic interactions. A complex H-bond network consists of intra- and interresidue intramolecular hydrogen bonds that affect the overall molecular structure: two interresidue bonds namely, IdoA2S_NR_(C–3)O–H···O–(C–3) GlcNS,6S_R_ hydrogen bond and the GlcNS,6S_R_ N–H···O(2–O–SO_3_^−^)IdoA2S_R_ were found in **1**. Neither of these bonds are present in **2** and intraresidue H-bonds are formed instead in this conformer. As each form (^1^*C*_4_ or ^2^*S*_0_) of the IdoA2S residue has a unique hydrogen bond configuration, formation of the ^2^*S*_0_ form must be accompanied by breaking both hydrogen bonds in **1** and forming two new intraresidue hydrogen bonds in **2**. These changes in the H-bond network must influence the conformational equilibrium and influence the internal dynamics of this tetrasaccharide in the aqueous solution. Theoretical data also indicate that water molecules from the first hydration shell are also involved in the H-bond network with various saccharide groups. The computed distances are compatible with both donor and acceptor weak bifurcated hydrogen bonds. Apart from H-bonds, the overall molecular structure of **1** and **2** is affected by ionic interactions with counterions.

The computed three-bond proton–proton coupling constants enabled determination of the conformer population in the solution: the chair ^1^C_4_ form of the IdoA2S residues is more populated (67%) than the skew form (33%) in the aqueous solution. The analysis of spin–spin coupling constants indicated that the Fermi–contact contributions to ^3^*J*_H–H_ coupling constants are affected by the oxygen lone pairs located in distinct locations within the tetrasaccharide molecule. The DFT–computed paramagnetic and diamagnetic spin–orbit terms, larger than the Fermi–contact contributions for H1–H2 coupling constants, are in agreement with our previous analysis and these terms depend upon geometrical factors arising from the contributions of localised molecular orbitals of adjacent residues. This is also compatible with the DSO term, which is smaller than the FC in the monosaccharide, IdoA2SOMe. Similarly, the FC and DSO terms are comparable to the IdoA2S_R_ residue in heparin disaccharide, indicating smaller contributions of its solitary neighbouring unit (GlcNS,6S_NR_). The data presented here, therefore, offer a detailed insight into the interpretation of spin–spin coupling constants and underline the application of appropriate quantum-chemical methods in the analysis of the solution properties of heparin oligosaccharides.

## Figures and Tables

**Table 1:** Selected optimised (B3LYP/6-311++G(d,p)) interatomic distances (in Å) and bond angles (in degrees) in the heparin tetrasaccharide. Two conformers of the IdoA2S residues are considered: ^1^*C*_4_ (**1**) and ^2^*S*_0_ (**2**). The GlcNS,6S residues are in the ^4^*C*_1_ form.

Residue	Bond	1	2
GlcNS,6S_NR_	C1-C2	1.546	1.531
	C1-O5	1.405	1.417
	C1-O1	1.411	1.415
	O1-C4_IdoA2S(NR)_	1.409	1.444
IdoA2S_NR_	C1-C2	1.544	1.540
	C1-O5	1.388	1.407
	C1-O1	1.451	1.424
	O1-C4_GlcN,6S(R)_	1.430	1.443
	C6-O51	1.270	1.256
	C6-O52	1.239	1.260
GlcNS,6S_R_	C1-C2	1.544	1.532
	C1-O5	1.416	1.422
	C1-O1	1.397	1.414
	O1-C4_IdoA2S(NR)_	1.434	1.436
IdoA2S_R_	C1-C2	1.540	1.539
	C1-O5	1.408	1.422
	C1-O1	1.418	1.431
	C6-O51	1.263	1.257
	C6-O52	1.248	1.256
GlcNS,6S_NR_	O5-C1-C2	113.4	109.0
	O5-C1-O1	112.4	111.0
	C1-O1-C4_IdoA2S(NR)_	123.3	117.0
IdoA2S_NR_	O5-C1-C2	115.2	115.1
	O5-C1-O1	113.2	113.4
	C1-O1-C4_GlcN,S(R)_	119.7	120.1
GlcNS,6S_R_	O5-C1-C2	108.7	109.1
	O5-C1-O1	113.5	111.2
	C1-O1-C4_IdoA2S(R)_	122.0	117.2
IdoA2S_R_	O5-C1-C2	113.2	112.2
	O5-C1-O1	112.3	109.3
	C1-O1-C_Me_	114.4	112.3

**Table 2:** Selected optimised (B3LYP/6-311++G(d,p)) torsion angles (in degrees) in the heparin tetrasaccharide. Two conformers of the IdoA2S residues are considered: ^1^*C*_4_ and ^2^*S*_0_ to provide, respectively, two forms of the heparin tetrasaccharide, (**1**) and (**2**). The GlcNS,6S residues are in the ^4^*C*_1_ form.

Residue	Torsion Angle	1	2
GlcNS,6S_NR_	O5-C1-C2-C3	48	64
	H1-C1-C2-H2	46	66
	H2-C2-C3-H3	−160	174
	H3-C3-C4-H4	161	165
	H4-C4-C5-H5	−170	−162
	H1-C1-O1-C4_IdoA2S(NR)_	−67	−39
IdoA2S_NR_	O5-C1-C2-C3	−36	12
	H1-C1-C2-H2	85	133
	H2-C2-C3-H3	−84	−144
	H3-C3-C4-H4	73	115
	H4-C4-C5-H5	59	47
	H4-C4-O1-C1_GlcN,6S(NR)_	−44	−48
	H1-C1-O1-C4_GlcN,6S(R)_	99	76
GlcNS,6S_R_	O5-C1-C2-C3	68	61
	H1-C1-C2-H2	67	61
	H2-C2-C3-H3	168	176
	H3-C3-C4-H4	176	165
	H4-C4-C5-H5	−161	−161
	H4-C4-O1-C1_IdoAS(NR)_	18	16
	H1-C1-O1-C4_IdoA2S(R)_	−38	−33
IdoA2S_NR_	O5-C1-C2-C3	−50	33
	H1-C1-C2-H2	72	153
	H2-C2-C3-H3	−75	−177
	H3-C3-C4-H4	70	141
	H4-C4-C5-H5	56	42
	H4-C4-O1-C1_GlcN,6S(R)_	−24	−46

**Table 3:** Intraresidue and interresidue hydrogen bonds in forms **1** and **2** of the heparin tetrasaccharide. Atoms involved in hydrogen bonds are in italics; distances are in Å.

Residue	Hydrogen Bonds–Intraresidue	1	2
GlcNS,6S_NR_	O*H*_(C–3)_···S*O*_3 (NS*O*3-)_	1.9	2.1
	O*H*_(C–4)_···6–*O*_(6-*O*-SO3-)_	2.1	2.2
	N*H*···1–*O*_(glycosidic)_	2.1	-
IdoA2S_NR_	O*H*_(C–3)_···S*O*_3 (2-O-S*O*3-)_	-	1.9
GlcNS,6S_R_	O*H*_(C–3)_···S*O*_3 (NS*O*3-)_	1.9	2.0
IdoA2S_R_	O*H*_(C–3)_···1–*O*_(-*O*-CH3)_	2.0	-
	O*H*_(C–3)_···2–*O*_(2-*O*-SO3-)_	-	2.2
	**Hydrogen bonds–Interresidue**		
	O*H*_(C–3)_ IdoA2S_NR_···*O*H _(C–3)_ GlcNS,6S_R_	1.9	-
	N*H* GlcNS,6S_R_···2–*O*_(2–*O*–SO3-)_ IdoA2S_R_	2.1	-

**Table 4:** Selected computed torsion angles and three-bond proton–proton coupling constants (values in Hz) in heparin tetrasaccharide. The two forms (**1** and **2**) correspond to different conformations (^1^*C*_4_ and ^2^*S*_0_) of the IdoA2S residues. The GlcNS,6S residues are in the ^4^*C*_1_ conformation. <^3^*J*_H-C-C-H_> was computed as a weighted average using data presented in columns 5 and 6 using the ratio 67:33 (**1**:**2**). Experimental values are shown in the last column.

Residue	Array of Atoms	Torsion Angles 1	Torsion Angles 2	^3^*J*_H-C-C-H_ 1	^3^*J*_H-C-C-H_ 2	<^3^*J*_H-C-C-H_> 67:33 (1:2)	Expt. *
GlcNS,6S_NR_	H1-H2	45.6	65.5	4.45	2.89	3.9	3.5
	H2-H3	−160.0	174.0	9.17	11.52	10.0	10.3
	H3-H4	160.6	164.7	8.97	9.47	9.1	9.7
	H4-H5	−169.9	−161.6	10.27	9.41	9.9	9.7
IdoA2S_NR_	H1-H2	85.2	133.2	1.19	3.95	2.1	2.9
	H2-H3	−83.7	−144.0	1.77	6.17	3.2	5.4
	H3-H4	73.3	114.9	3.27	2.34	3.0	3.8
	H4-H5	59.0	47.0	2.46	3.45	2.8	2.7
GlcNS,6S_R_	H1-H2	66.7	61.4	2.92	3.37	3.1	3.5
	H2-H3	168.3	175.6	10.33	12.32	10.9	10.3
	H3-H4	175.8	164.9	10.11	9.62	9.9	9.2
	H4-H5	−160.5	−161.1	9.54	9.31	9.5	9.2
IdoA2S_R_	H1-H2	72.4	152.8	1.50	6.00	3.0	2.9
	H2-H3	−74.7	−177.4	2.64	10.62	5.3	5.3
	H3-H4	70.0	140.6	3.49	3.58	3.5	3.9
	H4-H5	55.9	42.0	2.41	4.23	3.0	2.7

* Ref [28].

**Table 5:** DFT–computed (B3LYP/6-311+(d,p)) Fermi contact, spin–dipolar, paramagnetic spin–orbit and diamagnetic spin–orbit contributions to the three-bond proton–proton coupling constants (values in Hz) in form **1** of the heparin tetrasaccharide. Total ^3^*J*_H-C-C-H_ magnitudes are listed in the final column.

Conf. Residue	Array of Atoms	Torsion Angles	Fermi Contact	Spin–Dipolar	Paramgn. Spin–Orbit	Diamgn. Spin–Orbit	Total ^3^*J*_H-C-C-H_
GlcN,6S_NR_	H1-H2	45.6	3.66	0.13	−1.12	1.78	4.45
	H2-H3	−160.0	9.54	0.04	0.66	−1.07	9.17
	H3-H4	160.6	9.45	0.04	0.74	−1.26	8.97
	H4-H5	−169.9	10.64	0.04	0.63	−1.04	10.27
IdoA2S_NR_	H1-H2	85.2	0.32	0.04	−0.93	1.76	1.19
	H2-H3	−83.7	1.30	0.03	−0.71	1.15	1.77
	H3-H4	73.3	2.71	0.06	−0.96	1.46	3.27
	H4-H5	59.0	1.34	0.09	−1.73	2.76	2.46
GlcN,6S_R_	H1-H2	66.7	2.34	0.06	−0.92	1.44	2.92
	H2-H3	168.3	10.54	0.04	0.44	−0.69	10.33
	H3-H4	175.8	10.26	0.05	0.21	−0.41	10.11
	H4-H5	−160.5	9.56	0.03	0.09	−0.14	9.54
IdoA2S_R_	H1-H2	72.4	0.99	0.06	−0.67	1.12	1.50
	H2-H3	−74.7	2.24	0.04	−0.68	1.04	2.64
	H3-H4	70.0	3.07	0.07	−0.75	1.10	3.49
	H4-H5	55.9	1.64	0.12	−1.24	1.89	2.41

**Table 6:** DFT–computed (B3LYP/6-311+(d,p)) Fermi contact, spin–dipolar, paramagnetic spin-orbit and diamagnetic spin-orbit contributions to the three-bond proton–proton coupling constants (values in Hz) in form **2** of the heparin tetrasaccharide. Total ^3^*J*_H-C-C-H_ magnitudes are listed in the final column.

Conf. Residue	Array of Atoms	Torsion Angles	Fermi Contact	Spin–Dipolar	Paramgn. Spin–Orbit	Diamgn. Spin–Orbit	Total ^3^*J*_H-C-C-H_
GlcN,6S_NR_	H1-H2	65.5	2.32	0.08	−0.91	1.40	2.89
	H2-H3	174.0	11.86	0.04	0.67	−1.05	11.52
	H3-H4	164.7	9.86	0.04	0.71	−1.14	9.47
	H4-H5	−161.6	9.73	0.04	0.65	−1.01	9.41
IdoA2S_NR_	H1-H2	133.2	3.74	0.01	0.06	0.14	3.95
	H2-H3	− 144.0	6.19	0.03	0.21	−0.26	6.17
	H3-H4	114.9	2.27	0.01	−0.16	0.24	2.34
	H4-H5	47.0	2.51	0.12	−1.41	2.23	3.45
GlcN,6S_R_	H1-H2	61.4	2.75	0.09	−1.00	1.53	3.37
	H2-H3	175.6	12.61	0.05	0.55	−0.89	12.32
	H3-H4	164.9	9.81	0.04	0.37	−0.60	9.62
	H4-H5	−161.1	9.40	0.04	0.31	−0.44	9.31
IdoA2S_R_	H1-H2	152.8	6.30	0.04	0.71	−1.05	6.00
	H2-H3	−177.4	10.96	0.05	0.68	−1.07	10.62
	H3-H4	140.6	3.86	0.02	0.45	−0.75	3.58
	H4-H5	42.0	3.28	0.14	−1.42	2.23	4.23

**Table 7:** DFT–computed (B3LYP/6-311+(d,p)) Fermi contact, spin–dipolar, paramagnetic spin–orbit and diamagnetic spin–orbit contributions to three–bond H1–H2 proton–proton coupling constants (values in Hz) in monosaccharide IdoA2SOMe and in other structurally similar heparin–like oligosaccharides. Total ^3^*J*_H-C-C-H_ magnitudes are listed in the final column.

	Residue	Array	Torsion Angle	FC	SD	PSO	DSO	Total
Monosaccharide [21]	IdoA2SOMe	H1-H2	72	1.32	0.05	−0.45	0.74	1.67
Disaccharide [25]	IdoA2S_R_	H1-H2	73	0.98	0.05	−0.61	1.07	1.49
Trisaccharide [26]	IdoA2S_NR_	H1-H2	75	1.05	0.04	−1.07	1.70	1.72
Tetrasaccharide *	IdoA2S_NR_	H1-H2	85	0.32	0.04	−0.93	1.76	1.19
Pentasaccharide [27]	IdoA2S_NR_	H1-H2	64	1.90	0.08	−1.27	2.18	2.89

* This work.
